# Effect of compound dietary fiber of soybean hulls on the gel properties of myofibrillar protein and its mechanism in recombinant meat products

**DOI:** 10.3389/fnut.2023.1129514

**Published:** 2023-02-23

**Authors:** Song-Shan Zhang, Jun-Ya Duan, Teng-Teng Zhang, Meng Lv, Xiao-Guang Gao

**Affiliations:** ^1^Institute of Animal Sciences, Chinese Academy of Agricultural Sciences, Beijing, China; ^2^College of Food Science and Biology, Hebei University of Science and Technology, Shijiazhuang, Hebei, China

**Keywords:** compound dietary fibers, gel strength, water holding capacity, rheological properties, secondary structure, chemical force, microstructure

## Abstract

Response surface methodology was used to determine the optimum ratio of rice husk dietary fiber, soybean hull dietary fiber, and inulin as 1.40, 1.42, and 3.24%. The effects of compound and single dietary fiber on water holding capacity, gel strength, secondary structure, rheological properties, chemical action force, and microstructure of myofibrillar proteins (MP) gel were investigated. The application of composite dietary fiber significantly (*P* < 0.05) improved the gel strength, water holding capacity and storage modulus (G′) of MP gel. Fourier transform infrared spectrum analysis shows that the addition of compound dietary fiber can make the gel structure more stable. The effect of dietary fiber complex on the chemical action of MP gel was further studied, and it was found that hydrophobic interaction and disulfide bond could promote the formation of compound gel. By comparing the microstructure of the MP gel with and without dietary fiber, the results showed that the MP gel with compound dietary fiber had smaller pores and stronger structure. Therefore, the rice hull dietary fiber, the soybean hull dietary fiber and the inulin are compounded and added into the low-fat recombinant meat product in a proper proportion, so that the quality characteristics and the nutritional value of the low-fat recombinant meat product can be effectively improved, the rice hull dietary fiber has the potential of being used as a fat substitute, and a theoretical basis is provided for the development of the functional meat product.

## 1. Introduction

Fat is a very important component in recombinant meat products, helping to maintain the original taste, texture and appearance of meat and increasing satiety during meals. Fats change the perception of taste compounds by affecting the balance, intensity and release of taste as well as their distribution and migration. However, excessive fat intake increases the risk of obesity, certain cancers, and other diseases (1). In recent years, low-fat or non-fat meat products have become a hot research topic (2). But reducing fat in meat products can affect product appearance, flavor and texture (3). Many scholars have found that part of the fat can be replaced by non-meat components while offsetting the adverse effects of the low fat content due to its strong water holding capacity to (4). Studies have proved that dietary fiber can be used as a fat substitute with its excellent physical and chemical properties and functional characteristics (5).

The hulls of grains and beans contain a large amount of dietary fiber, which can cause a waste of resources if directly discarded (6, 7). At present, dietary fiber is mainly produced by physical methods (superfine grinding, high-pressure treatment, and extrusion), chemical methods (alkali method and acid method), and biological methods (fermentation method and enzyme method) (8, 9). Alkaline treatment is to use most of the impurities such as protein and starch can be dissolved in the alkali liquor, by controlling the appropriate reaction conditions to make dietary fiber. Studies have shown that alkali method can not only effectively produce dietary fiber, but also make part of the hemicellulose and cellulose hydrolysis modification into soluble dietary fiber. The cost of preparing dietary fiber by the alkali method is low, and the large-scale production is convenient to realize.

Inulin is derived from Jerusalem artichoke, onion and other plants, and is a natural water-soluble dietary fiber that can be added to meat products as a fat substitute (10). Generally, the gel-forming effect is good when the addition amount of inulin is more than 10 %, but excessive inulin can cause harm to the human body. Therefore, there is a need to maximize the quality of meat products while limiting the amount of inulin added. It is an effective solution to use multiple dietary fibers in combination to make them work synergistically. At present, there are few studies on the application of the combination of insoluble dietary fiber and soluble dietary fiber in meat products.

The quality of meat mainly depends on the properties of myofibrillar proteins (MP) (11). MP accounts for 55–60% of total muscle protein and 10–20% of skeletal muscle weight. It plays an important role in the water holding capacity, elastic properties and gel strength of meat products, and is an important structural protein affecting the quality of muscle food (12, 13). Therefore, the properties of MP were of great significance for studying the mechanism of meat products.

In this paper, the chaff and the soybean hull powder are treated by an alkali method to prepare the hull dietary fiber which is compounded with inulin, developing a low-fat recombinant meat product with high dietary fiber; by analyzing the effects of compound dietary fiber and single dietary fiber on protein components, gel properties, intermolecular chemical forces, the secondary structure and microstructure of protein of pork MP gel system, the interaction mechanism of compound dietary fiber and pork MP was explored in order to provide a theoretical basis for developing the application of dietary fiber in meat processing and improving the quality of low-fat recombinant meat products.

## 2. Materials and methods

### 2.1. The dietary fiber from chaff by alkaline method

The chaff and soybean hull materials were cleaned from impurities and placed in a blast dryer (210 mm, Huawei Chemical Instruments, Wuhan, Hubei, China) and dry overnight. The two raw materials were crushed by a crusher (FSJ302-5, Taist, Tianjing, China) and sieved by a 60-mesh sieve to obtain raw material powder. Each treated raw material powder was individually vacuum packed in polyethylene bags and stored in a −20°C refrigerator (BCD-251WP3CX, Changhong Meiling, Hefei, Anhui, China) for use. A certain amount of raw material powder was accurately weighed, added with 20 g/L NaOH solution in the solid to liquid ratio of 1:15 (g:mL), and placed in a water bath (HH-4, Shengwei, Shanghai, China) at 60°C for extraction for 40 min. The mixed system was filtered and the filtrate was washed with water to neutral; the supernatant was precipitated by adding 4 times the volume of anhydrous ethanol for 6 h. The filter residues were obtained by centrifugation (HC-3018, Zhongke Zhongjia, Anhui, China) at 1,274 × *g*, and the pH was adjusted to neutral. The two filter residues were mixed and freeze-dried to produce dietary fiber.

### 2.2. Response surface methodology (RSM)

Response surface methodology (RSM) was used to determine the effect of the three independent variables, that is, the addition amount of chaff dietary fiber(A), the addition amount of soybean hull dietary fiber (B), and the addition amount of inulin (C).

Seventeen treatments were conducted based on the Box-Behnken design (BBD). Each run was done in triplicate. The designed response surface tests are presented in Table 1.

**TABLE 1:** Response surface experimental design.

Test number	A (Chaff dietary fiber)	B (Soybean hull dietary fiber)	C (Inulin)
1	1.00% (−1)	1.00% (−1)	3.34% (0)
2	2.00% (+1)	1.00% (−1)	3.34% (0)
3	1.00% (−1)	2.00% (+1)	3.34% (0)
4	2.00% (+1)	2.00% (+1)	3.34% (0)
5	1.00% (−1)	1.50% (0)	2.50% (−1)
6	2.00% (+1)	1.50% (0)	2.50% (−1)
7	1.00% (−1)	1.50% (0)	4.17% (+1)
8	2.00% (+1)	1.50% (0)	4.17% (+1)
9	1.50% (0)	1.00% (−1)	2.50% (−1)
10	1.50% (0)	2.00% (+1)	2.50% (−1)
11	1.50% (0)	1.00% (−1)	4.17% (+1)
12	1.50% (0)	1.00% (−1)	4.17% (+1)
13	1.50% (0)	1.50% (0)	3.34% (0)
14	1.50% (0)	1.50% (0)	3.34% (0)
15	1.50% (0)	1.50% (0)	3.34% (0)
16	1.50% (0)	1.50% (0)	3.34% (0)
17	1.50% (0)	1.50% (0)	3.34% (0)

According to the preliminary experiment based on RSM, the optimum condition of the three dietary fibers was determined and was used in the following experiments, which was 1.40% chaff dietary fiber, 1.42% soybean hull dietary fiber, and 3.24% inulin, respectively.

### 2.3. Extraction of myofibrillar protein (MP)

Extraction of MP was carried out as described by Park et al. (14) with minor modifications. The longest muscle of pig back was thawed for 4 h at 4°C in advance, cleaned, and tidied, and the meat was cut into small pieces and weighed in a pre-cooled beaker. Buffer solution (containing 10 mmol/L Na_2_HPO_4_/NaH_2_PO_4_, 2 mmol/L MgCl_2_, 1 mmol/L EGTA, 0.1 mmol/L NaCl, 7.0) was added in a volume ratio of 1: 4, and the mixture was stirred evenly with a glass rod (for about 1 min) and homogenized at high speed for 2 min. The connective tissue was filtered out with two layers of sterile gauze. The samples were centrifuged at 5,000 × g for 15 min at 4°C, and the precipitate was collected. Repeat the above procedure three times to obtain the precipitate as crude MP. The crude MP and 0.1 mmol/L NaCl solution were mixed in a volume ratio of 1:4, and centrifuged (H2050R, Xiangyi, Changsha, Hunan, China) (consistent with the centrifugal conditions during extraction) for three times. The obtained precipitate was purified MP, which was stored under seal at 4°C and used up within 48 h. The whole preparation process is carried out at 4°C. Bovine serum albumin was used as the standard, and the extracted MP concentration was determined by biuret method.

### 2.4. Sodium dodecyl sulfate–polyacrylamide gel electrophoresis (SDS–PAGE)

According to the method described by Lv et al. (15) with minor modifications, conduct sodium dodecyl sulfate polyacrylamide gel electrophoresis (SDS-PAGE) to observe protein cross-linking and polymerization, the extracted MP was diluted to a certain concentration with the loading buffer. And then cooled to room temperature after a water bath of 100°C for 10 min to produce the electrophoretic loading sample. The MP was analyzed by SDS-PAGE gel electrophoresis (WD-9413D, Liuyi, Beijing, China) and imaged in the gel imaging system.

### 2.5. Preparation of dietary fiber-MP composite gels

The MP was dissolved (0.6 mol/L NaCl,50 mmol/L NaH_2_SO_4_/Na_2_HSO_4_, pH 6.2) in the extract to adjust the protein concentration to 40 mg/mL. Different dietary fibers were added, respectively, homogenized at a low speed for 30 s by a homogenizer (FSH-2, Mengte, Changzhou, Jiangsu, China), and placed in a constant temperature water bath, which was gradually heated from room temperature to 75°C at a rate of 1°C/min and kept in the 75°C water bath for 10 min before being quickly removed and cooled in ice for 30 min, and finally stored overnight in a 4°C refrigerator for backup. All samples were equilibrated at room temperature for 30 min before testing.

According to the optimal proportion of the composite dietary fiber in the low-fat recombinant meat product obtained by the response surface method. Hull dietary fiber 1.40%, soybean hull dietary fiber 1.42%, inulin 3.24%, the experimental groups were divided into five groups: the control group (T0); added 6.06% chaff dietary fiber (T1); added 6.06% soybean hull dietary fiber (T2); added 6.06% inulin (T3); added 1.40% chaff dietary fiber +1.42% soybean hull dietary fiber +3.24 % inulin (T4).

### 2.6. Gel strength

According to the method described by Zhuang et al. (16) with minor modifications, the gel samples were cut into cylinders with a height of 2 cm, and the fracture strength and fracture distance were measured on a physical property analyzer (TA. XTPlus, Supertechnical, Fujian, China) using a spherical probe (P/5s). The fracture strength is the value corresponding to the first highest peak on the puncture curve, and the distance at which this peak is formed is the fracture distance. The gel strength value is the product of fracture strength and fracture distance. Specific parameters were set as follows: automatic trigger type, induced force of 5.0 g, and test speed of 1.0 mm/s. The maximum sustain force is that gel strength.

### 2.7. Water holding capacity (WHC)

A certain mass of gel sample (designated as *W*_1_) was weighed, placed in a centrifuge tube (designated as *W*_0_), and centrifuged at 10,000 × *g* for 15 min at 4°C, and weighed the final mass of the sample after centrifugation (17). Three parallel experiments were conducted for each group, and the sampling position as consistent as possible. The water holding capacity of composite gel was calculated according to Formula (1-1):


$$\text{WHC}=\frac{w_{2}-w_{0}}{w_{1}-w_{0}}\times 100\%\,\left(1-1\right)$$

In the formula, *W*_0_—centrifugal tube quality(g); *W*_1_—quality before centrifugation(g); *W_2_—*total mass of tube and sample after centrifugation (*g*).

### 2.8. Rheological measurements

According to the method described by Sun et al. (18) with minor modifications to determine the rheological properties (Viscotester iQ, Thermo Fisher Scientific Shier, Guangzhou, China) of the samples. Tests were performed in gradient warming oscillation mode using 50 mm plates. The samples were uniformly coated on the test platform. Before the test, the sample edges were sealed with silicone oil to keep closed. Then, the temperature was increased from 20 to 80°C at a rate of 1°C/min with a frequency of 0.1 Hz and a plate spacing of 0.6 mm.

### 2.9. Secondary structures

The gel samples were freeze-dried and pulverized, and KBr was added for milling. KBr sample tablets were prepared under the pressure of 600 kg/cm^2^ and scanned in the range of 4,000 cm^–1^–500 cm^–1^ by using a Fourier transform infrared spectrometer (Nicolet6700, Thermo Fisher, USA) (19).

### 2.10. Determination of the intermolecular chemical forces

According to the method of Gómez-Guillén (20), weighing 2 g of the gel sample, adding 10 mL of S1 solution (0.05 mol/L NaCl, 10 ml), S2 solution (0.6 mol/L NaCl), S3 solution (0.6 mol/L NaCl + 1.5 mol/L urea), Solution S4 (0.6 mol/L NaCl + 8 mol/L urea), solution S5 (0.6 mol/L NaCl + 8 mol/L urea + 0.5 mol/L β- mercaptoethanol), homogenized at low speed for 2 min, placed in an environment at 4°C for 1 h, and centrifuged at 10,000 × *g* for 15 min at 4°C. The supernatant was collected, and the content of protein was determined by biuret method. The chemical acting force is calculated according to formulae 1-2)–1-5:


Ionic⁢bond=Protein⁢content⁢in⁢S2⁢solution-protein⁢content⁢in⁢S1⁢solution⁢(1-2)

Hydrogen⁢bond=Protein⁢content⁢in⁢S3⁢solution-Protein⁢content⁢in⁢S2⁢solution⁢(1-3)

Hydrophobic⁢interaction=Protein⁢content⁢in⁢S4⁢solution

-Protein⁢content⁢in⁢S3⁢solution⁢(1-4)

Disulfide⁢bond=Protein⁢content⁢in⁢S5⁢solution-Protein⁢content⁢in⁢S4⁢solution⁢(1-5)

### 2.11. Scanning electron microscopy (SEM) of gel structures

According to the method described by Zhuang et al. (21) with minor modifications, the gel samples were cut into small cubes (5 mm × 5 mm × 5 mm) and fixed in 2.5% glutaraldehyde solution at 4°C for 1 day under protection from light. The 0.1 mol/L treated gel samples were washed three times with phosphate buffer (pH 7.4) for 10 min each. Dehydration was performed sequentially with a gradient of ethanol solution (60, 70, 80, 90, and 100%) for 15 min each time. The gel samples were immersed in isoamyl acetate for 15 min and repeated twice, and finally the samples were freeze-dried for 24 h, fixed with double-sided conductive adhesive and then sprayed with gold. The microstructure of the treated gel samples was observed using scanning electron microscopy (S-4800-I, HITACHI, Tokyo, Japan) at 2,500× and 4,000× magnification.

### 2.12. Statistical analyses

The experiment of each group was repeated three times, and the final result was expressed as means ± standard deviation. The optimum process conditions were obtained by software Design-Expert V8.0.6.1. The data was processed by SPSS 21.0 software. Analyze data for plotting with Origin 2019, Omnic and PeakFit.

## 3. Results and discussion

### 3.1. Component analysis of the MP

**FIGURE 1:**
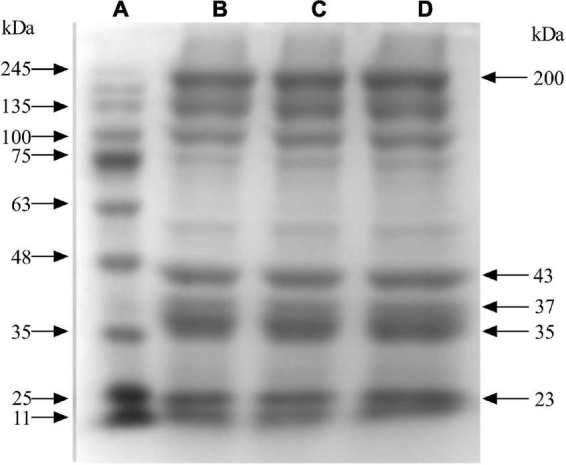
MP electrophoresis results. A is Marker; B, C, and D are MP.

Myofibrillar protein is the protein of muscle myofibrils, which is composed of myosin, tropomyosin, myosin, and actin (22). According to the electrophoresis results in Figure 1, the MP extracted in this test contained the basic components of MP, such as myosin heavy chain (200 kDa), actin (43 kDa), troponin (37 kDa), tropomyosin (34–36 kDa), and myosin light chain (16–25 kDa). The bands in each group were clear and complete, and the results were basically the same as those reported by Li (23), indicating that the extracted MP could be used for subsequent studies.

### 3.2. Gel strength

**FIGURE 2:**
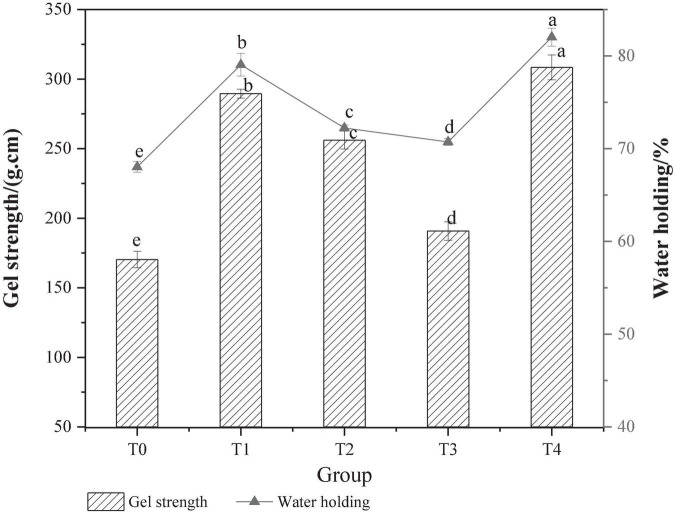
Effect of composite dietary fiber on MP gel strength and water retention. T0: control group; T1: 6.06% chaff dietary fiber; T2: 6.06% soybean hull dietary fiber; T3: 6.06% inulin; T4: 1.40% chaff dietary fiber +1.42% soybean hull dietary fiber +3.24% inulin; a–e: different letters indicated significant differences among the samples (*P* < 0.05).

Gel strength can reflect the gel-forming ability of protein, which is related to the gel network structure (24). The effect of different dietary fibers on the strength of MP gel is shown in Figure 2. Compared with the control T0 group, the gel strength was significantly increased in the T1 group added with chaff dietary fiber, the T2 group added with soybean hull dietary fiber, and the T4 group added with compound dietary fiber were increased significantly (*P* < 0.05). Based on the physicochemical properties of dietary fiber from gluten meal, it was speculated that the improvement of pork MP gel strength might be closely related to the strong water absorption and expansion of dietary fiber from gluten meal. During the thermal processing, the uniformly distributed dietary fibers are expanded and then filled into the MP gel network, resulting in the improvement of gel strength. Inulin has an effect on the gel strength of MP, which may be due to its own gel-forming property, making it possible to increase the number of gel molecules that can be formed per unit volume, strengthening the collision and cross-linking between dietary fiber and protein molecules, and the gel strength is improved (25). The adding effect of the composite dietary fiber is better than that of the single dietary fiber at the same concentration, probably due to the synergistic effect of the different types of dietary fiber on the MP gel, resulting in a significant increase in gel strength (*P* < 0.05).

### 3.3. WHC

The WHC of MP gel reflected the water binding capacity and gel degree of MP gel in the system, which had a very important effect on the quality of recombinant meat products (26). As shown in Figure 2, the WHC of the composite gel system was significantly (*P* < 0.05) improved with the addition of dietary fiber. The WHC of the T0 control group was (68.03 ± 0.58)%, and that of the T3 group added with inulin was (70.73 ± 0.36)%, which was 3.97 % higher than that of the T0 control group. This is because inulin can form a strong gel network structure with MP, blocking the free flow of water. The movement of the water molecules is constrained, and the water contained in the water molecules is not easily separated from the MP even under the action of an external force. Therefore, the WHC of the gel is greatly improved. Compared with other groups, the WHC of T4 group with compound dietary fiber was the strongest, which was (82.02 ± 0.97)%, increased by 17.62% compared with the control T0 group. This indicated that the addition of compound dietary fiber could promote the cross-linking of MP during the heat-induced gel process and significantly improve the water binding capacity of the final gel structure (27).

### 3.4. Rheological property

**FIGURE 3:**
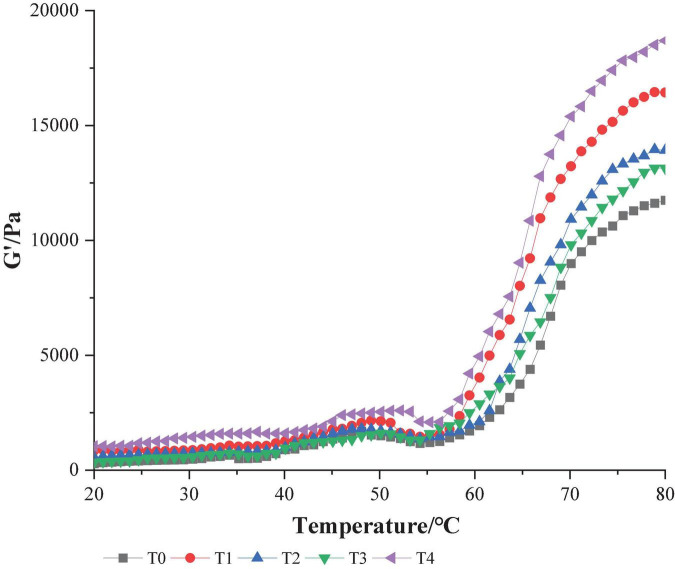
Effect of composite dietary fiber on rheological properties of MP gel. T0: control group; T1: 6.06% chaff dietary fiber; T2: 6.06% soybean hull dietary fiber; T3: 6.06% inulin; T4: 1.40% chaff dietary fiber +1.42% soybean hull dietary fiber +3.24% inulin.

Myofibrillar protein gel has unique viscoelasticity, and its physical and chemical properties can be reflected by rheology (28). The change of Storage modulus (G′) may reflect the expansion or aggregation of the protein with an increase in temperature, the process of forming an elastic gel network structure. A higher value of G′ indicates a stronger gel-forming ability of protein (19). As shown in Figure 3, the MP storage modulus G′ of dietary fiber increased when compared with that of the control group, and the storage modulus G′ of group T4 when dietary fiber was added was the largest. These results showed that compound dietary fiber could promote the formation of gel structure to the maximum extent. In the control group, the value of G′ gradually increased at 40–50°C, reached its peak at about 50°C and then gradually decreased, rising sharply at 55–80°C. This phenomenon reflected that the temperature began to rise and the protein gel network was initially formed. As the further increase of temperature, leading to the denaturation and recombination of protein molecules, the final stable protein gel is formed (29). The temperature at which the G′ value changed in the T4 group when dietary fiber was added was later than that in other groups, and it occurred at about 54°C. This might be because the three dietary fibers combined to produce a synergistic effect, fully absorbing water and swelling, blocking the development of protein molecules, leading to an increase in the denaturation temperature of protein. Wang et al. (30) believes that synergy between several different exogenous additives can significantly increase that G′ value of MP gel, resulting in higher gel strength, similar to the results of this test.

### 3.5. Changes of secondary structures

**FIGURE 4:**
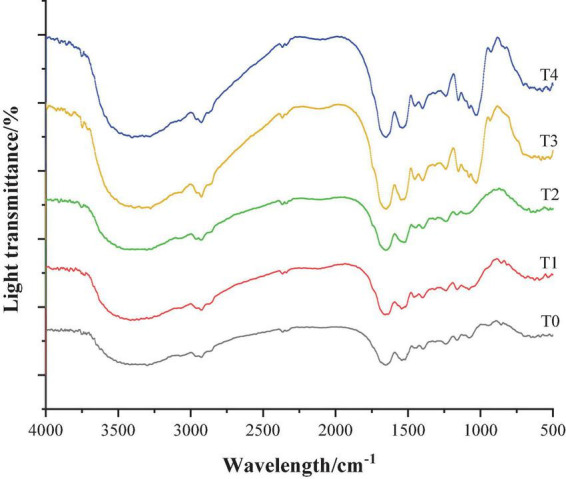
Infrared spectrum of composite dietary fiber-MP composite gel. T0: control group; T1: 6.06% chaff dietary fiber; T2: 6.06% soybean hull dietary fiber; T3: 6.06% inulin; T4: 1.40% chaff dietary fiber + 1.42% soybean hull dietary fiber + 3.24% inulin.

Fourier transform infrared spectrometer can selectively absorb infrared wavelengths and generate different characteristic absorption peaks according to the difference in the internal structure of the sample, so that the Fourier transform infrared spectrometer can be used for analyzing the change law of the secondary structure of the protein (31). Figure 4 shows the infrared spectrum of the dietary fiber-MP composite gel. The amide I-band was located in the range of 1,700–1,600 cm^–1^, and it characterized the C-O elastic vibration. The amide II band was located in the range of 1,600–1,500 cm^–1^, and characterized by N–H bending vibration and C–N extensional vibration. The amide A-band, located in the range of 3,600–3,200 cm^–1^, characterizes the O–H stretching vibration of water molecules, so it is also called “water zone” (32).

As shown in Figure 4, the infrared spectra of MP gel group added with dietary fiber were similar to those of T0 control group, indicating that the addition of dietary fiber would not change the protein skeleton structure. With the addition of dietary fiber, two new absorption peaks appeared in the infrared spectra of MP gel at 1,100–1,000 cm^–1^ and 3,700–3,600 cm^–1^, and the peak intensity of the group added with compound dietary fiber increased the most. In addition, compared with the control group, the addition of dietary fiber enhanced the peak intensity at 2,933 and 1,531 cm^–1^, and the wave number was slightly shifted.

The secondary structure of a protein is extremely sensitive to changes in the amide I band, so it can be used to analyze the secondary structure of a protein. The relationship between the secondary structure and the corresponding absorption peak was as follows: 1,650–1,660 cm^–1^ represented an α-helix structure, 1,600–1,640 cm^–1^ represented a β-fold structure, 1,660–1,695 cm^–1^ represented a β-turn and 1,640–1,650 cm^–1^ represented a random coil structure (33). The changes in the secondary structure of dietary fiber-MP composite gel are shown in Table 2. Compared with the MP gel of the control group, the α-helix and random coil contents of MP gel added with dietary fiber were decreased, while the β-sheet and β-corner contents were significantly increased. This indicates that the transformation from α-helix to β-fold and β-turn promotes the gelation of protein. In addition, in the α-helix content of MP gel decreased the most, while the β-sheet and β-corner contents increased the most in the compound dietary fiber group, which was conducive to the formation of a more stable gel structure of MP. This was consistent with the results reported by Liu (34). Therefore, the results of this study proved that the added of compound dietary fibers could effectively change the secondary structure of MP gel and enhance the gel-forming ability of protein, thereby improving the texture characteristics of recombinant meat products.

**TABLE 2:** Effect of composite dietary fiber on the secondary structure of MP gel.

Group	α-Helix	β-Fold	β-Rotation angle	Random coil
T0	41.68 ± 1.01^a^	27.83 ± 0.40^d^	17.29 ± 0.28^ac^	13.20 ± 0.92^a^
T1	34.24 ± 0.73^b^	39.04 ± 0.19^b^	18.51 ± 0.61^ab^	8.21 ± 0.57^c^
T2	35.42 ± 0.34^b^	36.90 ± 0.47^c^	17.41 ± 0.58^bc^	10.27 ± 0.40^b^
T3	31.84 ± 0.59^c^	40.49 ± 0.46^a^	16.68 ± 0.49^c^	10.98 ± 0.13^b^
T4	28.31 ± 0.54^d^	40.97 ± 0.47^a^	19.05 ± 0.71^a^	11.67 ± 1.14^ab^

T0: control group; T1: 6.06% chaff dietary fiber; T2: 6.06% soybean hull dietary fiber; T3: 6.06% inulin; T4: 1.40% chaff dietary fiber +1.42% soybean hull dietary fiber +3.24% inulin; Different letters in the same column indicated significant differences between the samples (*P* < 0.05).

### 3.6. Determination of the intermolecular chemical forces

**FIGURE 5:**
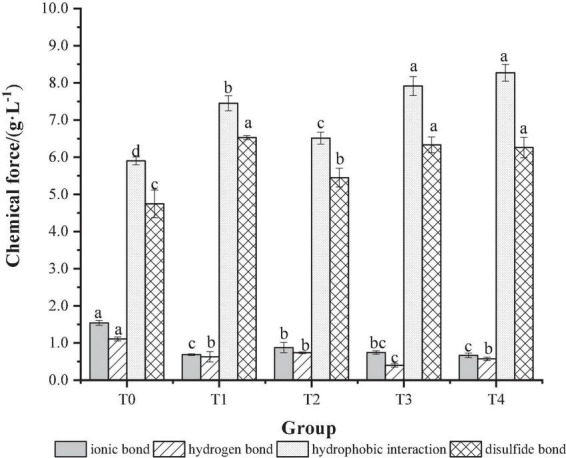
The effect of composite dietary fiber on the chemical force of MP gel. T0: control group; T1: 6.06% chaff dietary fiber; T2: 6.06% soybean hull dietary fiber; T3: 6.06% inulin; T4: 1.40% chaff dietary fiber + 1.42% soybean hull dietary fiber + 3.24% inulin. a–d: Different letters indicate that there are significant differences among the samples (*P* < 0.05).

The process of MP gel formation is related to chemical forces such as ionic bonds, hydrophobic interactions, hydrogen bonds and disulfide bonds, which together lead to changes in the gel structure of the protein and thus affect the gel properties of the protein (35). The effect of dietary fiber on the chemical action of MP gel is shown in Figure 5. As shown in Figure 5, compared with the T0 control group, the ionic bonds and hydrogen bonds of the group added with dietary fiber were significantly reduced (*P* < 0.05). This may be due to the consumption of hydrogen bonds during the transformation of the α-helix to β-fold during gel formation. The hydrophobic interaction and disulfide bond content were increased significantly (*P* < 0.05), suggesting that the hydrophobic interaction and disulfide bond could promote the formation of a more powerful and elastic three-dimensional network structure between protein molecules and promote the formation of gel (36). Compared with the T0 control group and the T1, T2, and T3 groups added with a single dietary fiber, the hydrophobic interaction and disulfide bond content of the T4 group added with the composite dietary fiber was higher, which indicates that there was a synergistic effect between the chaff dietary fiber, the soybean hull dietary fiber and inulin, which could improve the stability of the gel structure.

### 3.7. Scanning electron microscopy

**FIGURE 6:**
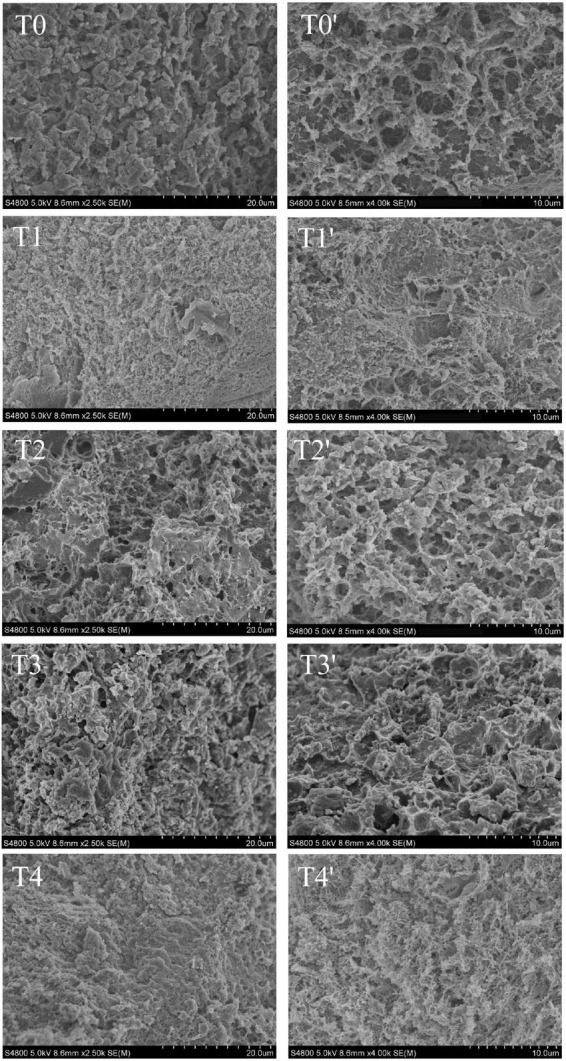
Effect of composite dietary fiber on the microstructure of MP gel. T0: control group; T1: 6.06% chaff dietary fiber; T2: 6.06% soybean hull dietary fiber; T3: 6.06% inulin; T4: 1.40% chaff dietary fiber + 1.42% soybean hull dietary fiber + 3.24% inulin. T0–T4: the sample is magnified by 2,500 times; T0′–T4′: the sample is magnified 4,000 times.

The network structure of MP gel had an important effect on the texture properties and water holding capacity of recombinant meat products (37). As shown in Figure 6, compared with the control group. The microstructure of pork MP gel added with dietary fiber was more compact, uniform and delicate. Compared with T0 control group, the gel structure of dietary fiber added is smaller, and the pores were decreased. Compared with the gel structure of the control group and the group added with the single dietary fiber, the structure is denser, the surface is smoother and the gap is smaller. This indicated that the protein structure formed by the combination of husk dietary fiber, soybean hull dietary fiber and inulin was superior to the protein gel structure of a single dietary fiber. The chaff dietary fiber and the soybean hull dietary fiber can play a filling role in the gel network. Inulin is uniformly dispersed in the gel structure without macropores. It can promote intermolecular covalent or non-covalent interactions, including disulfide bonds and hydrophobic interactions, can be promoted between protein-protein or protein-fibers. The inulin-MP mixed gel system formed a compact structure (38). The addition of inulin and dietary fiber from soybean hull into meat products could increase the total dietary fiber content and maintain the stability of the meat emulsion system. Therefore, the gel structure pores in the T1 and T2 groups were larger than those in the T4 group.

## 4. Conclusion

In this study, the compound dietary fiber composed of rice hull dietary fiber, soybean dietary fiber and inulin was added to meat products as a fat substitute. Response surface methodology was used to determine the optimum ratio of rice husk dietary fiber, soybean hull dietary fiber and inulin as 1.40, 1.42, and 3.24%. Compound dietary fibers can affect not only the network structure of MP gel, but also the chemical force between protein and change the gel characteristics. Compared with MP gel without dietary fiber and with single dietary fiber, the water holding capacity, gel strength and storage modulus G′ of the MP gel prepared with the compound dietary fibers added with chaff dietary fiber, soybean hull dietary fiber and inulin were significantly improved. Addition of complex dietary fibers promotes protein secondary structure in α- spiral direction β- fold and β- turn transformation, making the holes in the gel network smaller and the structure more uniformly dense. In addition, the addition of composite dietary fibers could reduce ionic and hydrogen bonds, increase hydrophobic interactions and disulfide bonds, and improve the stability of the gel structure.

In this study, it was found that the compound dietary fiber made of dietary fiber from chaff, dietary fiber from soybean hull and inulin affected the gel characteristics of pork MP, which was of great significance to improve the product quality. In the future, aiming at the changes in the tertiary structure of proteins, the mechanism of the effect of dietary fiber on low-fat recombinant meat products can be explored in depth, which will provide a theoretical support for the actual production.

## Instrument summary

Blast dryer (210mm, Huawei Chemical Instruments, Wuhan, Hubei, China), crusher (FSJ302-5, Taist, Tianjing, China), refrigerator (BCD-251WP3CX, Changhong Meiling, Hefei, Anhui, China), water bath (HH-4, Shengwei, Shanghai, China), high-speed centrifuge (HC-3018, Zhongke Zhongjia, Anhui, China), high-speed freezing centrifuge (H2050R, Xiangyi, Changsha, Hunan, China), electrophoretic gel imaging system (WD-9413D, Liuyi, Beijing, China), homogenizer (FSH-2, Mengte, Changzhou, Jiangsu, China), physical property analyzer (TA. XTPlus, Supertechnical, Fujian, China), rheometer (Viscotester iQ, Thermo Fisher Scientific Shier, Guangzhou, China), fourier infrared spectrometer (Nicolet6700, Thermo Fisher, USA), scanning electron microscopy (S-4800-I, HITACHI, Tokyo, Japan).

## Data availability statement

The original contributions presented in this study are included in the article/supplementary material, further inquiries can be directed to the corresponding author.

## Author contributions

X-GG and S-SZ conceived, designed the experiments, and supervised the study. ML performed the experiments and contributed to data curation. J-YD and T-TZ edited and wrote the manuscript. All authors contributed to the article and approved the submitted version.
